# Probiotic Properties of *Loigolactobacillus coryniformis* NA-3 and In Vitro Comparative Evaluation of Live and Heat-Killed Cells for Antioxidant, Anticancer and Immunoregulatory Activities

**DOI:** 10.3390/foods12051118

**Published:** 2023-03-06

**Authors:** Xiaoqing Xu, Yu Qiao, Qing Peng, Bo Shi

**Affiliations:** Feed Research Institute, Chinese Academy of Agricultural Sciences, Beijing 100081, China

**Keywords:** *Loigolactobacillus coryniformis* NA-3, probiotic, heat-killed, antioxidant, anticancer, RAW 264.7 macrophages

## Abstract

Some *Latiactobacilli* are often used as probiotics due to their functional activities, including antioxidant, anticancer and immunoregulation effect. *Loigolactobacillus coryniformis* NA-3 obtained from our laboratory is a promising probiotic according to the previous study. Coculture, the Oxford cup test and disk-diffusion methods were used to evaluate the probiotic properties and antibiotic resistance of *L. coryniformis* NA-3. The antioxidant activities of live and heat-killed *L. coryniformis* NA-3 were assessed via radicals’ scavenging ability. The potential anticancer and immunoregulatory capacity was determined in vitro using cell lines. The results indicate that *L. coryniformis* NA-3 has antibacterial activity and cholesterol removal ability and is sensitive to most antibiotics. Dead *L. coryniformis* NA-3 can scavenge free radicals as well as live strains. Live *L. coryniformis* NA-3 can significantly inhibit the proliferation of colon cancer cells; however, dead cells cannot. After RAW 264.7 macrophages were treated with live and heat-killed *L. coryniformis* NA-3, the production of NO, IL-6, TNF-α and reactive oxygen species (ROS) was induced. The increased expression of inducible nitric oxide synthase (iNOS) in treated macrophages mediates the production of NO. In conclusion, *L. coryniformis* NA-3 showed potential probiotic properties, and the heat-killed strain also exhibited activities similar to those of live bacteria, suggesting the possible value of its further application in the food processing and pharmaceutical industries.

## 1. Introduction

Lactic acid bacteria (LAB) are a group of lactic-acid-producing, Gram-positive, non-spore-forming, catalase-negative, anaerobic or microaerophilic, cocci or rods [1]. There are many species of LAB, including *Enterococcus*, *Lactobacillus*, *Pediococcus*, *Streptococcus*, *Lactococcus*, *Vagococcus*, *Leuconostoc*, *Oenococcus*, *Weissella*, *Carnobacterium* and *Tetragenococcus*, which are general microflora in both human and animal GITs [2]. Some LAB are commonly considered probiotics and generally recognized as safe (GRAS) [3]. Probiotics are defined by the Food and Agriculture Organization (FAO) of the United Nations and the World Health Organization (WHO) as “live microorganisms which when administered in adequate amounts confer a health benefit on the host” [4,5]. Probiotics can confer many benefits such as immunomodulation, lipid and cholesterol reduction, anticancer, antimicrobial and antioxidative properties, prevention of gastrointestinal infections, improvement of lactose metabolism, etc. [6,7]. The functional properties of a probiotic strain can be evaluated via various in vitro tests. The scavenging of free radicals is generally used for the in vitro evaluation of antioxidant activity, and cell experiments are effective methods of estimating biological activities such as anticancer and immunomodulation effects.

Reactive oxygen and free-radical species (ROFRs) are a group of highly reactive radicals and non-radical chemicals that can be produced during normal metabolism in the body and maintained at physiological levels by the work of endogenous enzymatic and non-enzymatic scavengers [8]. They fulfill various important biological functions such as cellular signaling, proliferation, apoptosis, regulation of gene expression and innate immunity to fight pathogens at suitable concentrations [8]. When ROFRs exceed the body’s own antioxidant capacity (AOC), they cannot be neutralized by the endogenous antioxidant system, which will lead to damage to the proteins, nucleic acids, lipids and carbohydrates of cells and tissues due to oxidation [9]. Ultimately, redundant ROFRs promote chronic diseases such as Parkinson’s disease, Alzheimer’s disease, atherosclerosis, arthritis, diabetes, neurodegenerative diseases, cardiovascular disease and cancer [8]. Therefore, it is necessary to clear harmful ROFRs to maintain the host’s health. Exogenous regulation and reasonable dietary antioxidant supplements are adopted means of controlling oxidative stress. Most LAB are equipped with scavenging systems for oxygen free radicals, possess antioxidant activity and can be used as alternatives for the production of functional foods or natural antioxidant supplements [10]. It is reported that viable cells of *Lactobacillus plantarum* 200655 isolated from kimchi can scavenge 1,1-diphenyl-2-picryl-hydrazyl (DPPH) and 2′-Azinobis-(3-ethylbenzthiazoline-6-sulphonate) (ABTS) free radicals and inhibit the oxidation of β-carotene and linoleic acid, showing significant antioxidant activity [11]. Intact cells of *Lactobacillus acidophilus* ATCC 4356 also show good antioxidative activity in terms of inhibiting linoleic acid peroxidation and scavenging DPPH free radicals [12]. Furthermore, both live and heat-killed *L. plantarum* Ln1 showed DPPH and ABTS scavenging activities and β-carotene bleaching inhibitory activity [13].

Studies have shown that live cells of probiotic bacteria have immune-potentiating effects [4]. For instance, *L. plantarum* BF_15 isolated from the feces of breast-fed infants could regulate intestinal microbiota dysbiosis in mice and expressed a potential immunomodulatory function [14]. Back in the 1900s, it was reported that *L. acidophilus* could promote the production of flavonoids by improving the activity of β-glucosidase, leading to antimutagenic, antioxidative and immune-stimulatory effects [15,16]. *Lactobacillus casei* CRL 431 administration in healthy mice increased the expression of Toll-like receptors (TLRs) and induced the secretion of cytokines, improving the immune response against *Salmonella enterica* serovar Typhimurium infection in mice [17]. Components of cell walls such as peptidoglycan (PG), lipoproteins (LP) and other proteins can be recognized by recognition receptors on the surface of host immune cells, activating the immune system [18]. Studies have shown that many functional effects obtained from viable cells of probiotics are also obtained from populations of dead cells [19], which may be closely related to the innate components of the bacterial cell wall. There have been reports that inactivated probiotic cells also stimulate the immune response via in vitro or in vivo evaluations. Nanometric heat-treated *L. plantarum* LM1004 isolated from Korean kimchi was shown to effectively induce the secretion of tumor necrosis factor-α (TNF-α) and interleukin 6 (IL-6) and to increase the expression of inducible nitric oxide synthase (iNOS) of macrophages in vitro [20]. Moreover, the oral administration of *L. plantarum* LM1004 also increased the splenocyte proliferation and serum cytokine levels in mice, suggesting good immunoregulatory activity in vivo [20].

Though the LAB used in the food, feed and pharmaceutical industries generally show many functional activities, there are still many issues to be solved. Live bacteria are easily degraded and mutated, hard to preserve, hard to transport, at risk for transfer of resistance genes in long-term use, likely to cause allergies and so on. Therefore, the development of novel probiotics is necessary. With the advancement of research, many functional activities similar to those of viable cells have been reported in dead LAB, which also represents an alternative method of solving the practical application problems of live bacteria. An exopolysaccharide (EPS)-producing *Latilactobacillus* was isolated from Chinese sauerkraut in our laboratory and was named *Lactobacillus coryniformis* NA-3. It has been known as *Loigolactobacillus coryniformis* since 2020, as its genus was reclassified. *L. coryniformis* almost never appears in various processing industries. However, our previous study showed that the EPS on the surface of *L. coryniformis* NA-3 exhibited antioxidant and biofilm-inhibiting properties [21]. This study focused on the further probiotic characteristics of the bacterial cell itself, including its antioxidant, anticancer and immune-enhancing activities. This study shows important research significance in terms of both probiotic development and solving the problem of live probiotics being used in applications.

## 2. Materials and Methods

### 2.1. Bacterial Strains and Culture Conditions

Foodborne *L. coryniformis* NA-3 was obtained from our own laboratory and isolated from Chinese sauerkraut. The bacteria stored at −80 °C was cultured in solid Man, Rogosa and Sharpe (MRS) (Solarbio, Beijing, China) broth at 37 °C for 48 h. A single colony of *L. coryniformis* NA-3 was transferred into 10 mL of MRS broth and grown at 37 °C for 48 h under anaerobic conditions. *Bacillus cereus* (CICC 21261), *Salmonella enterica* subsp. *enterica* serovar Typhimurium (CICC 22956/ATCC 14028) and *Escherichia coli* O157:H7 (CICC 10907) were purchased from the China Center of Industrial Culture Collection (CICC), and *Staphylococcus aureus* (CGMCC 1.291) was purchased from the China General Microbiological Culture Collection Center (CGMCC). The four pathogenic bacteria were grown in trypticase soy broth (TSB) solid medium at 37 °C for 24 h under aerobic conditions. A single colony was transferred into 5 mL of fresh TSB broth and grown for 24 h at 37 °C with shaking. A one-percent bacterial suspension (10^8^ CFU/mL) was inoculated in another 5 mL of fresh TSB medium and cultured for 24 h at 37 °C with continuous shaking in order to obtain a pathogenic bacteria suspension for the following applications.

### 2.2. Antibiotic Susceptibility

The disk diffusion method operates according to the principle that an antibiotic-impregnated disk placed on agar previously inoculated with the test bacterium picks up moisture, and the antibiotic diffuses radially outward through the agar medium, producing an inhibition zone [22]. In this study, the disk-diffusion method was used to evaluate the antibiotic susceptibility of *L. coryniformis* NA-3. Thirty antibiotic discs (BKMAM, Changde, China) were used in this study. The types, contents and judgment standards [23,24] of the antibiotics are listed in Table 1. The measurements were taken as follows. Antibiotic paper was placed on the surface of a Petri dish containing *L. coryniformis* NA-3 incubated at 37 °C for 24 h under anaerobic conditions. The diameter of the transparent circle was measured and expressed in millimeters.

### 2.3. Probiotic Properties of L. coryniformis NA-3

#### 2.3.1. Antibacterial Activity

##### Cocultivation

The antibacterial activity of *L. coryniformis* NA-3 was first assessed using the method of cocultivation with *B. cereus*, *S.* Typhimurium, *E. coli* O157:H7 and *S. aureus* according to the method described in our previous report [25]. The survival rate (%) of each pathogen was calculated as (CFU of pathogen in coculture system)/(CFU of pathogen in monoculture system) × 100%. CFU means colony-forming units.

##### Bacteriostatic Circle

The antibacterial ability of *Latilactobacillus* was measured using its supernatant and the Oxford cup method. *L. coryniformis* NA-3 was transferred into an 8 mL fresh MRS broth with 1% inoculation and grown for 24 h. The suspension was centrifuged at 10,000 r/min for 5 min. The supernatant was collected and used for the following bactericidal test. The pH of the supernatant was 4.0 after determination. *B. cereus*, *S.* Typhimurium, *E. coli* O157:H7 and *S. aureus* were also used for bacteriostatic circle tests. MRS medium (original pH 6.0) was used as a blank control. The supernatants of *L. coryniformis* NA-3 with the pH adjusted to 7.0 and MRS broth with the pH adjusted to 4.0 were also assessed to analyze the antibacterial effect. Petri dishes were placed at room temperature, and bacteriostatic tests were conducted on the following day.

#### 2.3.2. Cholesterol Removal and Bile Salt Hydrolase Production

The Oxford cup test was performed to identify the abilities of cholesterol degradation and BSH production using a Petri dish supplemented with 0.005% cholesterol. One hundred microliters of *L. coryniformis* NA-3 (10^9^ CFU/mL) was added into an Oxford cup, and MRS medium was used as the control. The Petri dish was incubated at 37 °C for 24 h under anaerobic conditions. The appearance of a white precipitating circle indicates cholesterol removal ability and BSH production. The white precipitate was mixed with ninhydrin solution and boiled for 5 min. A color reaction further confirmed the production of BSH.

### 2.4. Preparation of Live and Heat-Killed L. coryniformis NA-3 Cells

One milliliter of *L. coryniformis* NA-3 was inoculated in 100 mL of MRS medium and grown for 16 h at 37 °C under anaerobic conditions. The bacterial suspension was centrifuged at 12,000 rpm for 5 min. Cells were collected and washed twice with sterile phosphate-buffered saline (PBS, 0.01 M, pH 7.4). Finally, the precipitate was resuspended in sterile PBS and adjusted to 10^8^ CFU/mL as a sample of live strains for the next experiment. Half the volume of the bacteria suspension (10^8^ CFU/mL) was heat killed at 80 °C for 10 min in a water bath as a sample of heat-killed strains [26].

### 2.5. Observation of Live and Heat-Killed L. coryniformis NA-3 with Scanning Electron Microscope (SEM)

Live and heat-killed *L. coryniformis* NA-3 were observed using a Hitachi SU8010 scanning electron microscope (Hitachi, Tokyo, Japan) as described in our previous report [25].

### 2.6. Antioxidant Activity of Live and Heat-Killed L. coryniformis NA-3

#### 2.6.1. ABTS Scavenging

The ABTS-radical-scavenging activity assay using live and heat-killed *L. coryniformis* NA-3 was carried out as described by Song et al. [27], with a few modifications. The mixture of 400 μL of ABTS (Solarbio, Beijing, China) working solution (OD_734_ = 0.700 ± 0.02) and 100 μL of live/heat-killed strains was referred to as “Ai”. The mixture of 400 μL of ABTS solution (OD_734_ = 0.700 ± 0.02) and 100 μL of PBS (10 mM, pH 7.4) was referred to as “Ao”. All experiments were performed in triplicate. The mixtures were shocked and left for 6 min in the dark. Two hundred microliters of reaction supernatant was added to a 96-well plate after centrifugation (12,000 rpm for 5 min), and we measured the OD_734_ immediately.

The scavenging activity (%) = (OD_Ao_ − OD_Ai_)/OD_Ao_ × 100% (1)

#### 2.6.2. DPPH Scavenging

The scavenging activity of live and heat-killed *L. coryniformis* NA-3 on DPPH (MACKLIN, Shanghai, China) free radicals was determined as follows [27]. Suspensions of viable and heat-killed *L. coryniformis* NA-3 (10^8^ CFU/mL) were used for assessment, respectively. The mixture of live/heat-killed *L. coryniformis* NA-3 with DPPH was centrifuged at 12,000 rpm for 5 min, the supernatant of the reaction was added to a 96-well plate, and we measured the OD_517_. PBS was used as the negative control. Each experiment was performed in triplicate.

The scavenging activity (%) = (OD_negative control_ − OD_sample_)/(OD_negative control_) × 100% (2)

#### 2.6.3. Nitric Oxide Scavenging

The nitric oxide scavenging activity of the live and heat-killed *L. coryniformis* NA-3 was measured according to a modified version of the protocol described by Tsai et al. [28]. The supernatant of the reaction was determined at 550 nm. PBS was used as the negative control. Each experiment was performed in triplicate.

The scavenging activity (%) = (OD_negative control_ − OD_sample_)/(OD_negative control_) × 100% (3)

#### 2.6.4. Hydroxyl Radical Scavenging

The hydroxyl-radical-scavenging activity of live and heat-killed *L. coryniformis* NA-3 was evaluated according to our previous protocol, with a few modifications [29]. Live/heat-killed strains were added to the basic reaction system and referred to as “As”. PBS was mixed as “Ac”. The “Ao” group involved changing H_2_O_2_ and the sample to H_2_O and PBS. Each experiment was performed in triplicate. The supernatant of the reaction was added to a 96-well plate, and we immediately measured the optical density at 536 nm.

The scavenging activity (%) = (OD_As_ − OD_Ac_)/(OD_Ao_ − OD_Ac_) × 100% (4)

#### 2.6.5. Superoxide Free Radical Scavenging

The superoxide free radical scavenging of live and heat-killed *L. coryniformis* NA-3 was also determined using the method reported in [29], with a few modifications. The reaction system was prepared as follows: 100 μL of Tris (Solarbio, Beijing, China)-HCl (Beijing Chemical Works, Beijing, China) buffer (pH 8.0, 150 mM), 50 μL of 1,2,3-phentriol (Sinopharm Chemical Reagent Co., Ltd, Shanghai, China) (1.50 mM, dissolved with 10 mM HCl) and 200 μL of different samples referred to as “A11”. A mixture of Tris-HCl buffer, 10 mM of HCl and different samples was referred to as “A10”. A mixture of Tris-HCl buffer, 1,2,3-phentriol and PBS was referred to as “A01”. A mixture of Tris-HCl buffer, 10 mM HCl and PBS was referred to as “A00”. Each experiment was performed in triplicate. All reactions were vortexed and kept for 30 min in the dark at room temperature. After centrifugation (12,000 rpm for 5 min), 200 μL of supernatant was added to a 96-well plate, and then the OD_325_ was determined immediately.

The scavenging activity (%) = [1 − (OD_A11_ − OD_A10_)/(OD_A01_ − OD_A00_)] × 100% (5)

### 2.7. Anticancer Activity of Live and Heat-Killed L. coryniformis NA-3

HT-29 cells, purchased from Beijing Dingguo Changsheng Biotechnology Co., Ltd. (Beijing, China), were used to assess potential anticancer activity of live and heat-killed *L. coryniformis* NA-3. The cells were cultured as described in our previous work on macrophages [30]. Fifty thousand cells were seeded in a 96-well plate and grown overnight. HT-29 cells in a 96-well plate were treated with different dosages (10^6^ CFU/mL, 10^7^ CFU/mL and 10^8^ CFU/mL) of live/heat-killed *L. coryniformis* NA-3 in quadruplicate and cultivated for 24 h. After the cell supernatant was abandoned, the MTS method was used to determine the cell proliferation. The anticancer activity was measured via the proliferation result of HT-29 cells, which was expressed as percentage of OD_490_ relative to the negative control.

### 2.8. The Immunoregulatory Activity of Live and Heat-Killed L. coryniformis NA-3

RAW 264.7 macrophages were purchased from Beijing Dingguo Changsheng Biotechnology Co., Ltd and cultured using our previous method [30].

#### 2.8.1. Determination of Nitric Oxide

One hundred thousand cells in each well were seeded in a 96-well plate (100 μL/well) and grown for 24 h. A suspension (10^8^ CFU/mL) of live/heat-killed strains in PBS was centrifuged and resuspended in DMEM medium, supplemented with 10% FBS, and 1% penicillin–streptomycin, then diluted to 10^7^ CFU/mL and 10^6^ CFU/mL with DMEM medium containing 10% FBS. Then, the cells were treated with different concentrations of strains. Cells grown in complete DMEM medium were regarded as negative controls. All experiments were performed in quadruplicate.

After 24 h of treatment, the cultured supernatant was collected, and NO production was analyzed via a Griess assay [29]. The absorbance at 550 nm was read using a Synergy H1 microplate reader (BioTek, Winooski, VT, USA). The linear relationship between the concentrations of sodium nitrite and the absorbance was analyzed. The NO production was calculated based on the sodium nitrite standard curve.

#### 2.8.2. Determination of IL-6 and TNF-α

The IL-6 and TNF-α produced by macrophages treated with live/heat-killed *L. coryniformis* NA-3 was determined by the enzyme-linked immunosorbent assay (ELISA) method. We used ELISA Max™ Deluxe kits (BioLegend, San Diego, CA, USA), according to the manufacturer’s instructions.

#### 2.8.3. Proliferation

After collecting cell supernatant (Section 2.8.1), the MTS method was used for proliferation determination as described in Section 2.7.

#### 2.8.4. Intracellular Reactive Oxygen Species Measurement

ROS production in macrophages was quantified by the fluorescence spectrophotometry method described in our previous study [29]. A fluorescence intensity of 485/528 nm in each well was measured using a Synergy H1 microplate reader (BioTek, Winooski, VT, USA). The fluorescence intensity increased with the production of ROS. The result was calculated using the F485/528 result as follows:

ROS% = (The value of sample)/(The value of negative control) × 100%(6)

#### 2.8.5. The Expression of the iNOS of Macrophages Treated with Live and Heat-Killed *L. coryniformis* NA-3

RAW 264.7 macrophages (500,000 cells/well) were seeded in 6-well plates and grown overnight. The cells were treated with 10^7^ CFU/mL of live/ heat-killed *L. coryniformis* NA-3 and 10^8^ CFU/mL of heat-killed *L. coryniformis* NA-3 for 24 h. Treatment with LPS (1 μg/mL) was regarded as the positive control, and cells grown in DMEM medium with 10% FBS were the negative control.

##### Western Blot

After being treated for 24 h, the whole cell lysate was extracted, and Western blotting was performed according to our previous study [29]. The band intensity was quantitatively analyzed using ImageJ.

### 2.9. Statistical Analysis

Experiments were conducted at least in triplicate, and the results are presented as mean ± standard deviation. All statistical significances were analyzed using IBM SPSS Statistics Version 26 (IBM, Armonk, NY, USA). The data were evaluated with one-way ANOVA and compared using Duncan’s test. *p* < 0.05 was regarded as statistically significant.

## 3. Results and Discussion

### 3.1. Result of Antibiotic Susceptibility

Table 2 shows that *L. coryniformis* NA-3 was resistant to four antibiotics: oxacillin, imipenem, vancomycin and norfloxacin. The judgment standard of minocycline, azithromycin, lincomycin and florfenicol is not referred to at present. Although they had significant inhibition zones, they could not be defined. Another three antibiotics (ceftriaxone, kanamycin and ciprofloxacin) exhibited medium susceptibility for *L. coryniformis* NA-3. The other 19 antibiotics expressed sensibility for *L. coryniformis* NA-3. Antibiotic resistance genes can be transferred to other bacteria by conjugating plasmids and transposons [31]. It is hazardous to deliver antibiotic resistance genes to other microorganisms, especially pathogenic bacteria. Moreover, a sensitivity analysis of LAB is important when prescribing probiotic therapy against the background of the use of antibiotics. Our results suggest that *L. coryniformis* NA-3 cannot be used for therapy when most antibiotics are used, except for oxacillin, imipenem, vancomycin and norfloxacin. Reports suggest that *Latilactobacillus* is always sensitive to some antibiotics when used as a probiotic. William P Charteris reported that *L. acidophilus*, *L. casei*, *L. rhamnosus*, *L. reuteri* and *L. fermentum* were all sensitive to tetracycline, chloramphenicol, erythromycin and clindamycin when using the disc diffusion method [24]. These are similar to the results for *L. coryniformis* NA-3. However, *Latilactobacillus* does not exhibit broad-spectrum susceptibility to antibiotics. After being assessed via the disk diffusion test, *L. fermentum*, *L. crispatus*, *L. plantarum* and *L. jensenii* were mostly resistant to quinolones and showed moderate sensitivity to some other antibiotics [23]. The properties of *L. coryniformis* NA-3 are similar to those of *Latilactobacillus* as described in the literature, i.e., it expresses sensitivity to most antibiotics.

### 3.2. Probiotic Properties of L. coryniformis NA-3

#### 3.2.1. Antibacterial Result

**Figure 1:**
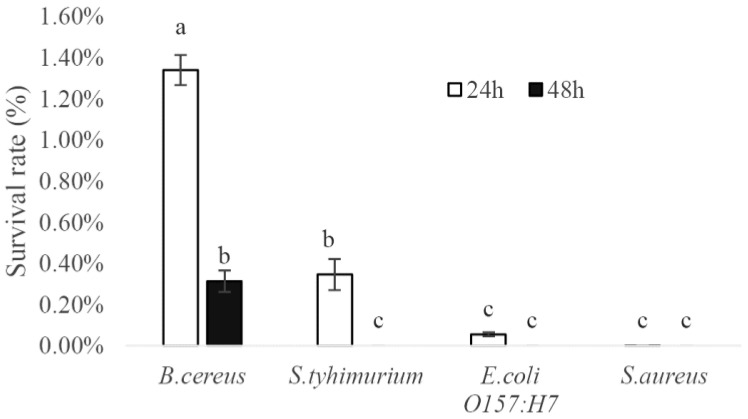
The survival rate of the four pathogenic bacteria (*B. cereus*, *S. typhimurium*, *E. coli* O157:H7, *S. aureus*) after being cocultured with *Loigolactobacillus coryniformis* NA-3 for 24 h and 48 h. Different lowercase letters mean significant differences (*p* < 0.05).

**Figure 2:**
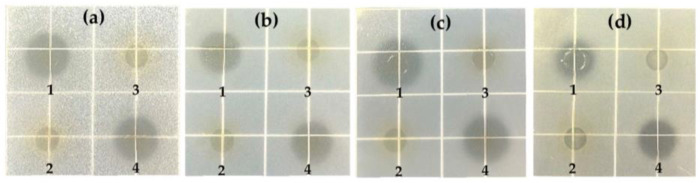
Antibacterial result of *Loigolactobacillus coryniformis* NA-3 supernatant evaluated by Oxford cup test. (**a**) *B. cereus*. (**b**) *S. Typhimurium*. (**c**) *E. coli* O157:H7. (**d**) *S. aureus*. 1: Original supernatant of *L. coryniformis* NA-3 (pH 4.0). 2: Supernatant of *L. coryniformis* NA-3 with pH adjusted to 7.0. 3: Original MRS broth (pH 6.0). 4: MRS broth with pH adjusted to 4.0.

The survival rates of *B. cereus*, *S. typhimurium*, *E. coli* O157:H7 and *S. aureus* after being cocultured with *L. coryniformis* NA-3 are shown in Figure 1. *B. cereus* had the highest survival rate of 1.34% at 24 h and 0.31% at 48 h. The survival rate of *S. typhimurium* (0.35%) was less than that of *B. cereus* (1.34%) at 24 h. After 48 h, *S. typhimurium* was undetectable; however, 0.31% of the *B. cereus* survived. *E. coli* O157:H7 and *S. aureus* were almost all dead at 24 h. These results suggest that *E. coli* O157:H7 and *S. aureus* were sensitive to *L. coryniformis* NA-3, and *S. typhimurium* was also sensitive to it but to a lesser extent. *B. cereus* exhibited some resistance to *L. coryniformis* NA-3. The supernatant of *L. coryniformis* NA-3 also exhibited antibacterial capacity in the four pathogenic bacteria, as Figure 2 shows. Generally, the antibacterial activity of probiotics should be confirmed under the condition of coexist for probiotic and pathogenic bacteria. Coculture is a means of determining the viability of microorganisms grown with other organisms. The results of cocultivation directly suggest that *L. coryniformis* NA-3 shows antibacterial activity. LAB generally show antibacterial activity, mostly due to their metabolites, such as organic acid (mostly lactic acid), bacteriocin and hydrogen peroxide. The antibacterial ability of metabolites is always evaluated with the supernatant of a bacterial suspension. The bacteriostatic circle method is commonly used to determine the antimicrobial activity of potential probiotics. *L. coryniformis* NA-3 supernatant showed no effect on the pathogenic bacteria after neutralization with alkali (Figure 2), and MRS broth supplemented with acid showed an obvious inhibition circle, suggesting that organic acid may be the main antimicrobial substance in the metabolites of *L. coryniformis* NA-3. The fermentation supernatant of *L. plantarum* zrx03 isolated from an infant’s feces strongly inhibited *E. coli* JM109, *S. aureus* and *Listeria monocytogenes*, and the inhibition zone was significantly observed [32]. The supernatant of *L. coryniformis* NA-3 acted on *B. cereus*, *S.* Typhimurium, *E. coli* O157:H7 and *S. aureus*, which showed similar inhibition zones, suggesting good antibacterial activity similar to that of *L. plantarum* zrx03.

#### 3.2.2. Cholesterol Removal and BSH Production Ability

**Figure 3:**
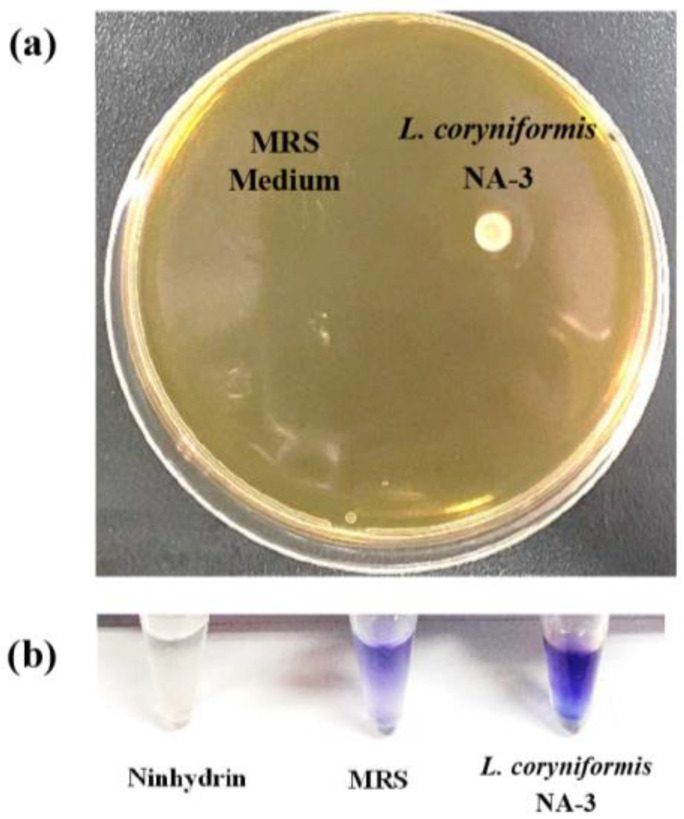
Result of cholesterol removal and bile salt hydrolase production of *Loigolactobacillus coryniformis* NA-3. (**a**) White sediment appeared when *L. coryniformis* NA-3 was grown on a medium supplemented with cholesterol. (**b**) The color reaction of white sediment and ninhydrin was obviously bluish-violet and darker than ninhydrin solution and MRS broth, indicating enzyme production.

The production of BSH is one of the assessment standards for probiotics. Bile salts can be decomposed into amino acids and cholic acids by BSH. A white precipitate is produced after cholic acids react with cholesterol. Thus, BSH-producing probiotics are helpful for cholesterol removal. White sediment appeared when *L. coryniformis* NA-3 was grown on a medium supplemented with cholesterol (Figure 3a). This phenomenon means that a reaction between cholic acid and cholesterol occurred, suggesting the production of BSH. The color reaction of white sediment and ninhydrin was obviously bluish-violet and darker than ninhydrin solution and MRS broth (Figure 3b), indicating enzyme production. It has been demonstrated that elevated serum cholesterol levels increase the risk of atherosclerosis and coronary heart disease [33]. Cholesterol removal is always used as an evaluation criterion for probiotics. Bile salt metabolism and cholesterol metabolism are closely related. Deconjugated bile salts by BSH are able to co-precipitate cholesterol at low pH levels, which is an effective in vitro experiment to expose the role of the BSH activity of *Latilactobacilli* in relation to cholesterol lowering in culture liquid [33]. *L. casei* ASCC 292 has been reported to exhibit cholesterol removal activity [34]. For *L. coryniformis* NA-3, similar results to *L. casei* ASCC 292 were observed. In addition, the ninhydrin reaction is a method for determining the production of enzymes. Results involving white sediment and color reactions suggest the cholesterol removal ability and BSH production of *L. coryniformis* NA-3.

### 3.3. Observation of Live and Heat-Killed Strains

**Figure 4:**
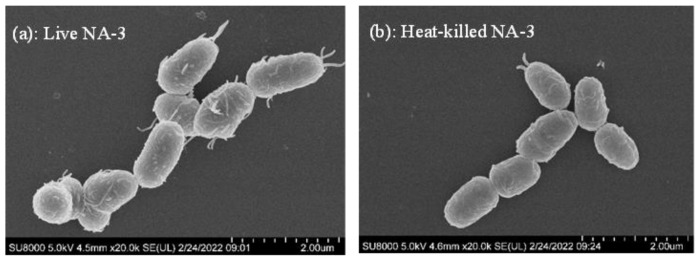
Micromorphology of live (**a**) and heat-killed (**b**) *Loigolactobacillus coryniformis* NA-3.

The cells of viable and heat-killed *L. coryniformis* NA-3 were observed with SEM, and the images are shown in Figure 4. *L. coryniformis* NA-3 was short and rod-shaped, presenting the typical characteristics of lactobacilli. Wirelike objects were attached to the surface of viable *L. coryniformis* NA-3 (Figure 4a). The attachments were significantly reduced after thermal killing, and they almost disappeared (Figure 4b). However, the integral morphology was not destroyed following heat treatment. Host–microbial interactions are complex molecular mechanisms. When bacteria arrive, host immune cells recognize the conserved microbial components, called “microbial–associated molecular patterns (MAMPs)”, through germline-encoded pattern recognition receptors (PRRs), activating innate defense mechanisms [35]. There are some MAMPs on the surface of probiotics, including lipopolysaccharide (LPS), peptidoglycan (PG), lipoteichoic acid (LTA), lipoprotein (LP) and others, which can specifically bind to Toll–like receptors–2 (TLR2) on intestinal cells [35]. As the images show, the cell wall of heat-killed *L. coryniformis* NA-3 maintained its original dimensional morphology. There were reserved active substances in cell walls, similar to viable bacteria, which may lead to the preservation of some of the biological functions of living *L. coryniformis* NA-3 cells in heat-killed cells.

### 3.4. Antioxidant Activity

**Figure 5:**
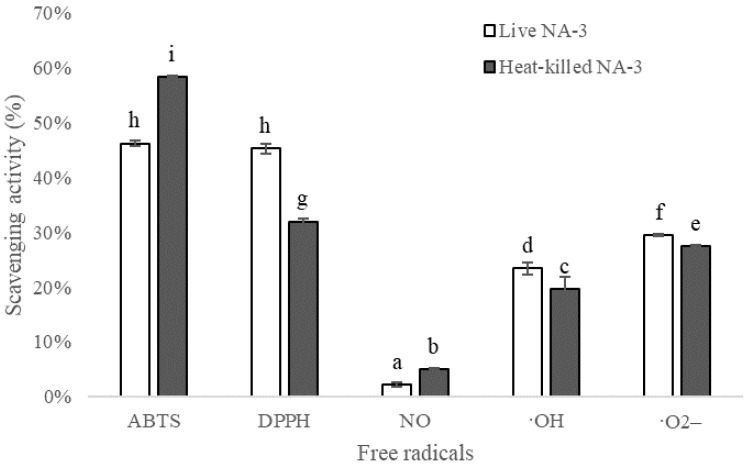
Antioxidant activity of live and heat-killed *Loigolactobacillus coryniformis* NA-3 evaluated by scavenging free radicals. Different lowercase letters mean significant differences (*p* < 0.05).

The antioxidant activity of live and heat-killed *L. coryniformis* NA-3 was evaluated by scavenging free radicals, as Figure 5 shows, including ABTS, DPPH, NO, hydroxyl radical, and superoxide radical. The results suggest that both live and heat-killed *L. coryniformis* NA-3 exhibited various scavenging capacities for different free radicals. Viable *L. coryniformis* NA-3 almost did not work on NO; however, heat-killed cells could scavenge a bit of NO (5%), showing better activity than live cells. *L. coryniformis* NA-3 can significantly scavenge the free radicals of ABTS, DPPH, hydroxyl radical and superoxide radicals. The scavenging ratio of heat-killed *L. coryniformis* NA-3 (58%) was also about 12% higher than that of viable cells (46%) on ABTS. Untreated *L. coryniformis* NA-3 was more active than heated cells when scavenging DPPH (45%), hydroxyl radicals (23%) and superoxide radicals (29%). Nonetheless, heat-killed cells also had different effects on scavenging DPPH (32%), hydroxyl radicals (20%) and superoxide radicals (27%), to varying degrees. In conclusion, *L. coryniformis* NA-3 shows antioxidant activity and can also maintain this capacity after thermal treatment. Heat-killed *L. coryniformis* NA-3 may be even better than live cells at radical scavenging in some instances.

It is known that antioxidative compounds on the cell surface are mostly proteins, exopolysaccharides and lipoteichoic acid. Antioxidant enzymes, bioactive peptides and manganese ions that exist in LAB cells also influence their antioxidant effects [11]. A previous study found that the intact cells of *L. plantarum* MA2 possessed a higher radical scavenging capacity than the culture supernatant and cell–free extract [9]. DPPH and ABTS scavenging activities are generally used to evaluate the antioxidant properties of live or killed *lactobacillus*. Seo-Jin Yang et al. [11] reported that viable *L. plantarum* 200655 can scavenge 32% of DPPH and 38% of ABTS free radicals. The live *L. coryniformis* NA-3 used in our study had higher scavenging activity on DPPH (45%) and ABTS (46%) than the reported *L. plantarum* 200655. Additionally, live/heat-killed *L. plantarum* Ln1 from Kimchi and *L. plantarum* KCTC 3108 were also effective at scavenging DPPH and ABTS [13]. For DPPH, *L. coryniformis* NA-3 (both live and heat-killed cells) showed higher scavenging activity than Ln1 and KCTC 3108. *L. coryniformis* NA-3 was weaker than Ln1 and KCTC 3108 at ABTS scavenging. In contrast, heat-killed *L. coryniformis* NA-3 was significantly better than live *L. coryniformis* NA-3 when it came to ABTS scavenging, which is different from the reported Ln1 and KCTC 3108. Additionally, our results also suggest that live and heat-killed *L. coryniformis* NA-3 exhibited NO, hydroxyl radical and superoxide free radical scavenging activities and that heat-killed cells in particular were more effective than live *L. coryniformis* NA-3 when it came to NO scavenging.

### 3.5. Anticancer Activity

**Figure 6:**
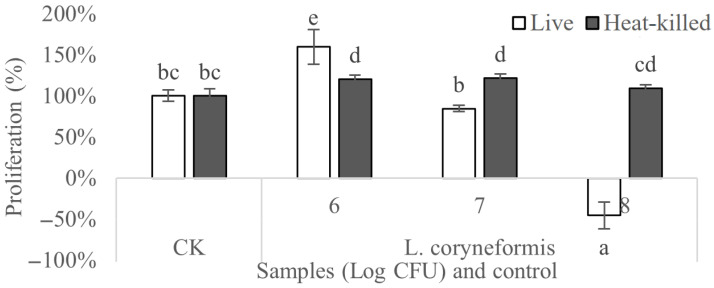
The anticancer activity of live and heat-killed *Loigolactobacillus coryniformis* NA-3 as evaluated using HT-29 cells. CK: Macrophages without treatment. Numbers 6, 7 and 8 on the abscissa: macrophages treated with different bacteria counts of Log CFU. Different lowercase letters mean significant differences (*p* < 0.05).

The anticancer activity of live and heat-killed *L. coryniformis* NA-3 was assessed using colon cancer cell HT-29 (Figure 6). The proliferation of HT-29 cells treated with heat-inactivated *L. coryniformis* NA-3 was not significantly different from the negative control, indicating no effect on the growth of cancer cells. Live *L. coryniformis* NA-3 cells obviously inhibited the proliferation of HT-29 cells with dose dependency. Cells grown with 10^6^ CFU/mL *L. coryniformis* NA-3 were all ineffective at the inhibition of HT-29 cell growth. The proliferation of cancer cells treated with 10^7^ CFU/mL (85%) live *L. coryniformis* NA-3 was significantly decreased when compared with untreated HT-29 cells (100%). HT-29 cells almost could not grow at all when cultivated with 10^8^ CFU/mL of live *L. coryniformis* NA-3. These results indicate that inactive *L. coryniformis* NA-3 has no inhibitory activity on the growth of cancer cells; however, live bacteria are effective. In vitro studies have demonstrated that live cells of probiotics possess higher antimutagenic activity by improving the host’s immune response, altering the metabolic activity of the intestinal microbiota, binding and degrading carcinogens, producing antimutagenic compounds and altering the physiochemical conditions in the colon [36]. Our results revealed that live *L. coryniformis* NA-3 was effective in terms of its anticancer effects, which may be attributed to some metabolites secreted from the viable strains. The reason that heat-killed *L. coryniformis* NA-3 cells were not effective in terms of cancer cell proliferation may be their inability to produce anticancer metabolites. It is reasonable to suppose that the metabolites of *L. coryniformis* NA-3 play an important role in its anticancer activity. However, multiple factors may be involved.

### 3.6. Immunoregulation Activity

#### 3.6.1. Proliferation

**Figure 7:**
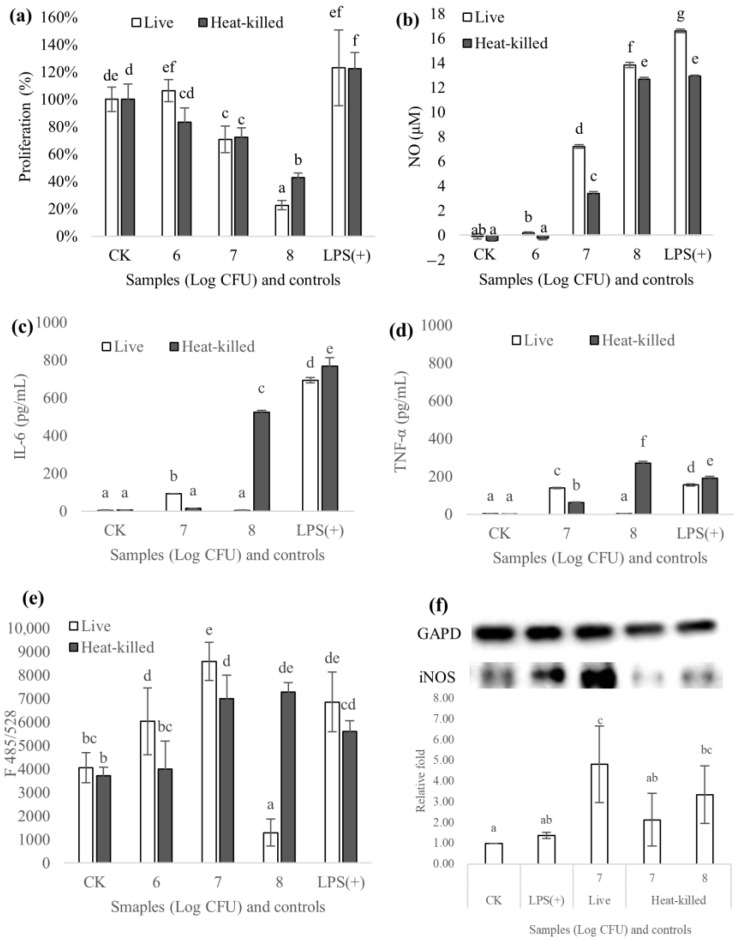
Results of proliferation (**a**), NO (**b**), IL-6 (**c**), TNF-α (**d**), ROS (**e**), and iNOS expression (**f**) of RAW 264.7 macrophages treated with live and heat-killed *Loigolactobacillus coryniformis* NA-3. Different lowercase letters mean significant differences (*p* < 0.05). CK: macrophages without treatment. LPS: macrophages treated with LPS (1 μg/mL). Numbers 6, 7 and 8 on the abscissa: macrophages treated with different bacteria counts of Log CFU.

A quantitative assay of proliferation was conducted using the MTS method, and the results are exhibited in Figure 7a. Compared to the untreated cells (100%), the proliferation of LPS-treated macrophages was promoted. *L. coryniformis* NA-3 showed dose-dependent cytotoxicity on macrophages, especially live strains. The proliferation of cells treated with 10^6^ CFU/mL of viable and heat-killed *L. coryniformis* NA-3 showed no significant difference when compared with the negative control (100%), indicating no cytotoxicity. The survivability of macrophages cocultured with 10^7^ CFU/mL live and heat-killed *L. coryniformis* NA-3 was 70% and 72%, showing no significant difference. *L. coryniformis* NA-3 at 10^8^ CFU/mL expressed serious cytotoxicity due to the proliferation of macrophages. The survivability of macrophages treated with 10^8^ CFU/mL of viable *L. coryniformis* NA-3 was only 23%. This value was 43% for the heated samples. Heat-killed *L. coryniformis* NA-3 had less of an impact than live samples at 10^8^ CFU/mL. The proliferation of treatments with 10^7^~10^8^ CFU/mL of samples demonstrated that heat-killed *L. coryniformis* NA-3 was less detrimental to cell reproduction than live samples and had higher security. A previous report showed that RAW 264.7 cell viability decreased as the concentration of *L. rhamnosus* GG and *L. brevis* KCCM 12203P increased. Additionally, viable *L. rhamnosus* GG and *L. brevis* KCCM 12203P exhibited lower cell viability than heat-killed cells, which is a similar result to that of our study [27].

#### 3.6.2. Production of NO and Cytokines (IL-6 and TNF-α)

A NO assay performed on RAW 264.7 cells was used to evaluate the immune-modulating properties of live and heat-killed *L. coryniformis* NA-3 in vitro (Figure 7b). Both viable and heat-killed samples can induce NO expression in macrophages. For *L. coryniformis* NA-3, the untreated and heat-killed cells were insufficient at 10^6^ CFU/mL to induce NO production. When strains were increased to 10^7^~10^8^ CFU/mL, NO was dramatically improved. The treatment groups of live *L. coryniformis* NA-3 produced 7.2 μM and 13.8 μM of NO at 10^7^ CFU/mL and 10^8^ CFU/mL, respectively. The production of macrophages induced by 10^7^ CFU/mL and 10^8^ CFU/mL of heat-killed *L. coryniformis* NA-3 were 3.4 μM and 12.7 μM NO, respectively, which are less than the values for viable cells. When the dosage of *L. coryniformis* NA-3 was increased to 10^8^ CFU/mL, the different effects of live and heat-killed samples decreased markedly. The data show that heat-treated *L. coryniformis* NA-3 also had significant immune-regulation properties that are similar to those of viable strains. It has been reported that live *lactobacillus* possesses immunomodulation characteristics. For example, *L. plantarum* 200655 isolated from cabbage kimchi enhanced immunity in RAW 264.7 cells by promoting the production of NO and cytokines [10]. Heat-killed *L. plantarum* emerged as a potentially important modulator of immune response, just as *L. plantarum* LM1004 enhances early innate immunity by activating macrophages via the TLR-2/MAPK/NF-κB signaling pathway [20]. The moderate release of NO in organisms can cause many physiological effects, such as promoting the activity of natural killer (NK) cells, activating peripheral blood mononuclear cells and regulating T lymphocytes. The excessive or insufficient production of NO produces a series of pathological effects that harm human health, e.g., by destroying DNA and some synthetases. Though NO performs a pivotal role as an immune regulator in a variety of tissues [37], it should be kept within appropriate limits. In this study, we demonstrated that *L. coryniformis* NA-3 was an effective strain for the NO production of macrophages, suggesting the potential stimulation of immunoregulation. Viable bacteria proliferation may be the main reason causing higher NO production when compared with heat-killed strains. Considering the safety evaluation, heat-killed probiotics may be a better immune activator for applications.

To survey the effect of live and heat-killed *L. coryniformis* NA-3 on immunoregulation, the production of IL-6 and TNF-α was also evaluated using ELISA. IL-6 and TNF-α were stimulated after cell exposure to live and heat-killed *L. coryniformis* NA-3. The production of IL-6 (Figure 7c) in the untreated RAW 264.6 cells was almost 0 pg/mL, whereas the cell supernatant cocultured with 1 μg/mL LPS was measured at 693~768 pg/mL. IL-6 in the cell culture that included heat-treated *L. coryniformis* NA-3 at 10^7^ CFU/mL and live strains at 10^8^ CFU/mL was almost undetectable, and no significant differences were detected between them and the negative group (0 pg/mL). However, the stimulation of IL-6 was increased to around 90 pg/mL after being co-induced with 10^7^ CFU/mL live samples. Furthermore, IL-6 in cell supernatant treated with heat-killed *L. coryniformis* NA-3 at 10^8^ CFU/mL was approximately 200 pg/mL lower than that of the LPS-stimulated group, which is an acknowledged immunomodulator activator. TNF-α production induced by different samples is illustrated in Figure 7d. Untreated RAW 264.7 macrophages and cells treated with 10^8^ CFU/mL of live *L. coryniformis* NA-3 almost did not secrete TNF-α. The production of TNF-α in cell supernatant co-stimulated with 10^7^ CFU/mL live *L. coryniformis* NA-3 was 138 pg/mL, which is obviously more than that observed in the heated strains (61 pg/mL). Heat-treated samples at 10^8^ CFU/mL expressed a stronger capacity for TNF-α induction (271 pg/mL) which was even significantly higher than the LPS control (155~190 pg/mL). Above all, both live and heat-killed *L. coryniformis* NA-3 revealed the stimulation of proinflammatory cytokines, suggesting immunoregulatory activity. Less production of IL-6 and TNF-α in live 10^8^ CFU/mL *L. coryniformis* NA-3 may be mostly due to its toxicity for cell proliferation. IL-6 and TNF-α are two major cytokines secreted by activated macrophages. These results suggest that *L. coryniformis* NA-3 promotes immunoregulatory effects via stimulating macrophages.

Macrophages are immune cells that play a crucial part in innate and adaptive immune responses. When macrophages are activated, they can produce NO to kill antigens and secrete cytokines such as IL-6 and TNF-α to activate specific immune responses. Many live and heat-killed lactobacilli have been reported to possess immune-enhancing activity by inducing NO and modulatory cytokine profiles in macrophages. For instance, *Lactobacillus fermentum* DLBSA204 can activate macrophage cells by improving NO and cytokine expression levels [38]. Live and heat-killed *L. rhamnosus* ATCC 7469 were effective at producing IL-6 and TNF-α in macrophage culture supernatants, suggesting the positive activity of strain *L. rhamnosus* ATCC 7469 in the modulation or in stimulation of immune responses [39]. The TNF-α production by macrophages cocultured with live *L. rhamnosus* ATCC 7469 is higher than that in heat-killed cells, which is consistent with *L. coryniformis* NA-3. IL-6 production is higher in macrophages after treatment with heat-killed *L. rhamnosus* ATCC 7469 when compared with live cells, which is similar to the result of 10^8^ CFU/mL of live and heat-killed *L. coryniformis* NA-3. NO, IL-6 and TNF-α were also effectively induced when macrophages were treated with both live and heat-inactivated *L. casei* and *L. plantarum* cells [40]. For different LAB, live and heat-killed strains show various activities when it comes to inducing NO and cytokines. Our results suggest that live *L. coryniformis* NA-3 has a better ability to secrete NO and cytokines (IL-6 and TNF-α). However, 10^8^ CFU/mL of live *L. coryniformis* NA-3 greatly inhibited cell proliferation, which is not a beneficial phenomenon. When considering cell cytotoxicity, heat-killed LAB may be safer for further applications.

#### 3.6.3. Reactive Oxygen Species

As Figure 7e shows, intracellular ROS in macrophages could be induced by *L. coryniformis* NA-3. It exhibited no significant differences in ROS production at 10^6^ CFU/mL with untreated cells. However, live *L. coryniformis* NA-3 expressed a little more potential than the heat-killed treatment. With an increase in dosage, the ROS production of cells treated with 10^7^ CFU/mL of live and inviable strains rises, especially for viable bacteria, showing better effect than LPS. At a dose of 10^8^ CFU/mL, cells treated with live strains only produce rare ROS, mostly due to cell cytotoxicity. However, 10^8^ CFU/mL of inactivated strains could also stimulate ROS production in macrophages that was almost not significantly different from LPS treatment. The ROS production of macrophages treated with live and heat-killed *L. coryniformis* NA-3 was significantly different. Live *L. coryniformis* NA-3 can grow and produce metabolites, but heat-killed strains cannot. It is reasonable to speculate that the mechanism for ROS production may be different for live and heat-killed *L. coryniformis* NA-3. Regardless, both live and heat-inactivated *L. coryniformis* NA-3 possessed the ability to induce ROS production. High concentration of ROS may lead to non-specific damage to proteins, lipids and nucleic acids, increasing the risk of cardiovascular disease, neurological disorders, cancers and chronic inflammation [41]. Additionally, ROS also play a critical role in various biological functions, including cell survival, cell growth, proliferation and differentiation and immune response [42]. Clearly, 10^6^~10^8^ CFU/mL of live *L. coryniformis* NA-3 and 10^7^~10^8^ CFU/mL of heat-treated strains have potential immune regulation activity via the inducing of the production of ROS. However, more ROS were generated by macrophages treated with live *Latilactobacillus* than those treated with inactivated samples at the same dose. In consideration of the risk to health of excessive ROS, heat-killed *L. coryniformis* NA-3 may be more suitable for use for immunoregulation.

#### 3.6.4. Expression of iNOS

Figure 7f shows the result of iNOS expression. Induced nitric oxide synthase (iNOS) expression was examined based on the production of NO (Figure 7a) generated by RAW 264.7 cells. *L. coryniformis* NA-3 enhanced the iNOS expression in macrophages both with and without heat treatment. Compared to LPS (1 μg/mL), 10^7^ CFU/mL of live *L. coryniformis* NA-3 significantly promoted iNOS production. However, the heat-killed strain was slightly weaker than live *L. coryniformis* NA-3. Three nitric oxide synthase (NOS) isozymes are responsible for NO production, including endothelial NOS (eNOS), neural NOS and inducible Ca^2+^/NOS (iNOS) [43]. The former two are Ca^2+^-dependent and are expressed constitutively (cNOS). The iNOS expression is Ca^2+^-independent and can provide a high output of NO generation for host defense, once induced [44]. Because NO is induced through the oxidation of L-arginine by iNOS, the production of iNOS cocultured with LAB strains showed similar results to NO production [11]. Previous reports have shown that *L. rhamnosus* GG, *L. plantarum* 200655 and *L. plantarum* KCTC 3108 all induced the expression of iNOS, which is consistent with the NO production and exerts immune-enhancing effects by stimulating macrophages [11]. Similar to the reported LAB (*L. rhamnosus* GG, *L. plantarum* 200655 and *L. plantarum* KCTC 3108), *L. coryniformis* NA-3 can also promote the NO production of macrophages via the NO/iNOS signal pathway and participate in the process of immune regulation.

## 4. Conclusions

*L. coryniformis* NA-3 isolated from Chinese sauerkraut showed susceptibility to most antibiotics, together with antibacterial and cholesterol removal properties. In addition, heat-killed *L. coryniformis* NA-3 also exhibited antioxidant and immunity-stimulation characteristics similar to those of live strains, which is promising for applications in functional food and the pharmaceutical industry as a safer potential probiotic and immuno-stimulating ingredient.

## Figures and Tables

**Table 1:** Types, names, contents, and judgment standards of 30 kinds of antibiotics.

Type	Name	Contents/Pcs	Judgement Standard/mm		
			Resistant (R)	Moderately Susceptible (MS)	Susceptible (S)
Group 1 Inhibitors of cell wall synthesis					
β-lactam	Oxacillin	1 μg			
	Penicillin	10 U	≤19	20–27	≥28
	Ampicillin	10 μg	≤12	13–15	≥16
	Piperacillin	100 μg	≤18	19–20	≥21
	Imipenem	10 μg	No standard		
Cephalosporins	Cephalexin	30 μg	≤14	15–16	≥17
	Cefamezin	30 μg	≤15	16–17	≥18
	Cefuroxime	30 μg	≤15	16–17	≥18
	Ceftazidime	30 μg	≤15	16–17	≥18
	Ceftriaxone	30 μg	≤13	14–20	≥21
	Cefoperazone	75 μg	≤15	16–18	≥19
Glycopeptides	Vancomycin	30 μg	≤14	15–16	≥17
Group 2 Inhibitors of protein synthesis					
Aminoglycosides	Amikacin	30 μg	≤15	16–17	≥18
	Gentamicin	10 μg	≤12	-	≥13
	Kanamycin	30 μg	≤13	14–17	≥18
	Streptomycin	10 μg	≤11	12–14	≥15
Tetracyclines	Doxycycline	30 μg			
	Tetracycline	30 μg	≤14	15–18	≥19
	Minocycline	30 μg	No standard		
Single antibiotics	Chloramphenicol	30 μg	≤13	14–17	≥18
Macrolides	Erythromycin	15 μg	≤13	14–17	≥18
	Azithromycin	15 μg	No standard		
Lincosamides	Clindamycin	2 μg	≤8	9–11	≥12
	Lincomycin	2 μg	No standard		
Amides	Florfenicol	30 μg	No standard		
Group 3 Inhibitors of nucleic acid synthesis					
Sulfonamides	Compound Sulfamethoxa	25 μg	≤10	11–15	≥16
Quinolones	Norfloxacin	10 μg	≤13	14–18	≥19
	Ciprofloxacin	5 μg	≤13	14–18	≥19
	Levofloxacin	5 μg	≤13	14–18	≥19
Group 4 Inhibitors of cytoplasmic membrane synthesis					
Polymyxins	Polymyxin B	300 IU	≤8	9–11	≥12

**Table 2:** Inhibition zone diameter (mm) and susceptibility results of antibiotics against *Loigolactobacillus coryniformis* NA-3.

Oxacillin	Penicillin	Ampicillin	Piperacillin	Imipenem
-(R)	38 (S)	37 (S)	36 (S)	-(R) *
Cephalexin	Cefamezin	Cefuroxime	Ceftazidime	Ceftriaxone
22 (S)	25 (S)	25 (S)	27 (S)	15 (MS)
Cefoperazone	Vancomycin	Amikacin	Gentamicin	Kanamycin
29 (S)	-(R)	18 (S)	18 (S)	16 (MS)
Streptomycin	Doxycycline	Tetracycline	Minocycline	Chloramphenicol
15 (S)	38 (S)	35 (S)	44 *	41 (S)
Erythromycin	Azithromycin	Clindamycin	Lincomycin	Florfenicol
40 (S)	35 *	40 (S)	12 *	42 *
Compound Sulfamethoxa	Norfloxacin	Ciprofloxacin	Levofloxacin	Polymyxin B
25 (S)	-(R)	14 (MS)	21 (S)	23 (S)

* No judgement standards.

## Data Availability

All methods and related data are presented in this paper. Additional inquiries should be addressed to the corresponding author.
